# Establishing Expectancy Values for Fibrin Monomer in Uncomplicated Pregnancy

**DOI:** 10.1055/s-0044-1788281

**Published:** 2024-07-11

**Authors:** Holger Seidel, Melina Duncklenberg, Hans-Jörg Hertfelder, Christine Gnida, Philipp Westhofen, Anna Stremlau, Joffrey Feriel, François Depasse, Hannah L. McRae, Johannes Philipp Kruppenbacher

**Affiliations:** 1 Centrum für Blutgerinnungsstörungen und Transfusionsmedizin, Bonn, Germany; 2 Clinical Development, Diagnostica Stago, Asnières sur Seine, France; 3 Institute of Experimental Hematology and Transfusion Medicine, University Hospital Bonn, Germany

**Keywords:** fibrin monomer test, D-dimer, pregnancy, postpartum period, thrombophilia

## Abstract

**Background** During pregnancy, a physiological increase of molecular activation markers (MAM) of hemostasis such as prothrombin fragments 1 + 2, thrombin–antithrombin complex, and D-dimers (DD) occurs. Therefore, monitoring MAM levels during pregnancy to evaluate the risk of venous thromboembolism (VTE) may be unreliable; nevertheless, DD analysis in pregnancy is widely performed. In contrast to DD, fibrin monomer (FM) levels have been reported to remain stable during pregnancy.

**Objectives** The main aim of this study was to define the expected range for FM levels in pregnant outpatients. In addition, we examined the impact of the individual VTE risk, as calculated by the pregnancy risk score of the Royal College of Obstetricians and Gynaecologists (RCOG), as well as that of antithrombotic treatment on FM levels.

**Methods** A total of 342 pregnant women seen at our hemostasis unit were included throughout 350 pregnancies in 899 samples.

**Results** Low-risk thrombophilia, but not the RCOG score itself, was found to influence all MAM levels, whereas antithrombotic treatment had only an impact on DD. For FM, a reference range could be calculated irrespective of the pregnancy term, in contrast to other MAMs, which fluctuated throughout pregnancy.

**Conclusions** Our findings suggest a stronger impact of inherited thrombophilia on hemostasis activity during pregnancy as compared with acquired or other predisposing thrombophilic risk factors. FM levels showed a marginal increase during pregnancy in contrast to other MAM and remain a potential candidate to improve the laboratory assessment of VTE risk during pregnancy. Further prospective studies in pregnant patients with suspicion of VTE are needed.

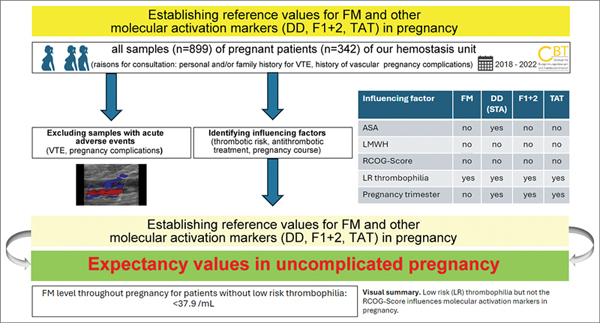

## Introduction

During pregnancy, a physiological increase in hemostasis activation occurs, leading to a hypercoagulable and hypofibrinolytic state to protect the fetomaternal barrier from hemorrhage and limit peri- and postpartum blood loss. 1 This is a continuous physiological process involving increases in fibrinogen, factors II, V, VII, VIII, IX, X, and XI, and von Willebrand factor, as well as partial decreases of anticoagulant components (e.g., protein S [PS]), and the downregulation of fibrinolysis via continuous increase of placenta-derived plasminogen activator inhibitor type 2 (PAI-2). 2 3 The secretion of PAI-2 into the maternal circulation is sufficient to inhibit the increasing concentration of tissue-type plasminogen activator, i.e., the most important plasminogen activator. This process leads to increased thrombin generation, which can be indirectly measured via molecular activation markers (MAM) such as prothrombin fragments 1 + 2 (F1 + 2), thrombin–antithrombin complexes (TAT), and D-dimer (DD). 4

Aside from substantial anatomical changes, this physiological shift toward a hypercoagulable state contributes to an increased risk of venous thromboembolism (VTE) in pregnancy, which continues to gradually increase 5- to 10-fold until delivery 5 6 7 and 15- to 35-fold in the puerperium 6 7 8 in comparison to nonpregnant women of comparable age. Assessment of the VTE risk in pregnancy is essential for optimum prevention and treatment of obstetric-associated VTE. The individual risk of VTE during and following pregnancy is dependent on the personal and/or family history of VTE, as well as the presence of inherited (e.g., factor V Leiden [FVL] or prothrombin G20210A mutation [PGM] and deficiencies of natural inhibitors antithrombin [AT], protein C [PC], and PS, etc.) and acquired risk factors (e.g., cesarean section, peri- and postpartum bleeding, etc.). Performing MAM (in particular DD) testing is often regarded as an important component for determining VTE risk during pregnancy and has been a widespread practice for decades, although this is not explicitly recommended by guidelines. 9 10 11 12 13 14 This individual laboratory-based approach may meet the recommendation of clinical surveillance in asymptomatic carriers of inherited thrombophilia but fails to predict VTE reliably. Furthermore, the detection of unexplained increased levels of MAM could lead to unnecessary further diagnostic evaluation and be disconcerting to the pregnant patient.

In nonpregnant patients, measurement of MAM is a routine component of VTE diagnosis, and DD is the most widely used and readily available of the MAM tests. The interpretation of DD results is challenging in pregnancy, during which successively increasing amounts of fibrin are formed due to hormone-associated changes causing increased vascular barrier permeability and subsequent leakage of plasma from capillaries into the surrounding tissue. The extravascular fibrin is then broken down by plasmin; this results in the creation of fibrin degradation products (FDP), including soluble DD complexes, which form specifically as a product of cross-linked fibrin degradation. Importantly, the DD assay cannot distinguish between intra- and extravascularly degraded fibrin. Other MAM such as F1 + 2 and TAT appear in the cascade prior to DD formation and could therefore be more representative of an active procoagulant process and thus predict VTE more reliably. F1 + 2 and TAT are being increasingly used in routine diagnostics and have been shown, for example, to yield a higher diagnostic value than DD for assessing VTE following total knee arthroplasty 15 ; however, these tests are often only available in specialized laboratories, and there is currently only one manufacturer that provides reagents for both tests. Furthermore, these tests only detect increased prothrombin–thrombin conversion and thrombin neutralization by AT, without detecting actual fibrin formation. This gap has recently been closed by the availability of automated latex immunoturbidimetric assays (LIA) for the detection of soluble fibrin, also known as fibrin monomers (FM).

FM is a marker of thrombin action on fibrinogen, in which thrombin cleavage removes the fibrinopeptides A and B from fibrinogen, 16 creating soluble FM. FM then binds to fibrinogen or FDP, forming noncovalently associated soluble FM complexes, which can be detected in plasma.

As previously reported, FM levels—in contrast to the aforementioned MAM—appear to be relatively stable throughout early and mid-pregnancy and only slightly elevated during late pregnancy. 17 18 To date, only a few studies have investigated FM in pregnancy via LIA, specifically the Auto LIA FM kit (Nissui Pharmaceutical Co Ltd, Tokyo, Japan) 17 19 and STA-LIATESTFM (Diagnostica Stago, Asnières sur Seine, France). 20 21 22 23

In a previous study, Grossman et al concluded that FM and DD are influenced by various maternal and obstetric factors and not solely gestational age, and that these parameters should therefore be considered when ruling out VTE in pregnancy. 22 The aim of our prospective study was to describe in detail hemostasis activation during pregnancy, evaluate the impact of maternal clinical characteristics such as thrombophilia and/or antithrombotic therapy on hemostasis, and establish reference values for FM, F1 + 2, TAT, and DD in pregnant patients. By addressing the current lack of reference ranges for these markers (including for different DD assays and the new STA-Liatest FM [Diagnostica Stago]) in pregnancy, we aim to provide better insight into the hypercoagulable shift observed in pregnancy as well as determine which MAM and corresponding laboratory tests could be most useful for assessing the VTE risk in this population.

## Methods

### Participants

Pregnant women ≥ 18 years of age who presented in our outpatient hemostasis unit for hemostasis testing between 2018 and 2022 were included. All patients had been referred because of personal or family history of VTE, vascular pregnancy complications (e.g., recurrent miscarriage, preeclampsia, premature placenta abruption, etc.), recurrent implantation failure, and/or known thrombophilic risk factors. The frequency of sample collection was not determined by the study design but was individually decided on a case-by-case basis to ensure that study sample collection would not interfere with patient care. We assessed the VTE risk during pregnancy by calculating the RCOG score, including dispositional and expositional risk factors. 11

### Ethics Approval

This study was approved by the Medical Ethics Committee of the Ärztekammer Nordrhein, Dusseldorf, Germany. Written informed consent was obtained from all subjects prior to their participation in the study.

## Materials and Methods

Venous blood samples were drawn and anticoagulated using S-Monovette tubes (Sarstedt, Nümbrecht, Germany), 109 mmol/L trisodium citrate (3.2%) with 1 vol. citrate + 9 vol. whole blood. Platelet-poor plasma was obtained after centrifugation of citrated whole blood for 15 minutes at 2,500 g. Approximately 1 hour following centrifugation, samples were aliquoted and immediately stored at −40°C for up to 2 months prior to sample analysis. FM and DD (DD STA) were measured on the Diagnostica Stago Compact Max Analyzer (Diagnostica Stago, Asnières sur Seine, France). DD were additionally performed via VIDAS D-Dimer Exclusion II (DD VIDAS) on a bioMérieux mini VIDAS ELISA system (both from bioMérieux, Marcy-l'Étolie, France) and D-Dimer HS 500 (DD HS) on ACL Top 750 CTS system (both from Werfen, Barcelona, Spain), respectively. F1 + 2 and TAT were quantified by ELISA using Enzygnost immunoassays (Siemens Healthineers, Marburg, Germany) on the Euroimmun Analyzer I (Euroimmun AG, Lübeck, Germany). The diagnosis of inherited thrombophilia risk factors (heterozygous/homozygous FVL and prothrombin G2023A mutation as well as deficiencies in the natural inhibitors AT, PC, and PS) were confirmed by molecular analysis.

### Statistics

The statistical analysis was performed using SPSS Statistics 28.0 (SPSS, Chicago, Illinois, United States) and RSoftware with RStudio (Posit Software, PBC formerly RStudio, Boston, Massachusetts, United States). For the identification of influencing parameters, we used nonparametric tests (Kruskal–Wallis test for global comparison and Wilcoxon test for comparison between two groups), as data were not normally distributed and failed to meet homogeneity of variance. *p* -Values below 0.05 (*) were considered statistically significant.

## Results

A total of 342 women were included throughout 350 pregnancies, from which a total of 899 samples were obtained and analyzed. The clinical characteristics of the study population are shown in Table 1 . The mean age ± 2 standard deviation (SD) was 32 ± 4.8 years (range: 21–48 years), with a mean prepregnant body mass index (BMI) ± 2 SD of 27.5 ± 6 kg/m ^2^ (range: 16–47 kg/m ^2^ ). A total of 143 of 342 (42%) patients underwent at least three examinations during their pregnancies (three: *n* = 37, four: *n* = 38, five *n* = 33, and ≥ six: *n* = 35). The average number of examinations per pregnancy was 2.6. Blood coagulation markers were measured at various time points throughout pregnancy including the first (week 1–12, *n* = 231), second (week 13–27, *n* = 337), and third trimesters (week 28–≥40, *n* = 241) as well as in the postpartum period (day 10–70, median days 40 postdelivery, *n* = 90).

**Table 1:** Maternal baseline characteristics and preexisting conditions of all 342 pregnant patients

	All patients ( *n* = 342)	RCOG score < 3 ( *n* = 195)	RCOG score ≥ 3 ( *n* = 147)	History of VTE ( *n* = 91)	History of pregnancy complications ( *n* = 141)
Maternal age (y ± 2 SD)	32 ± 4.8	32 ± 4.8	33 ± 5	33 ± 5	33 ± 5
Age > 35 y	87 (25%)	44 (23%)	43 (29%)	28 (31%)	46 (33%)
BMI (kg/m ^2^ ± 2 SD)	27.5 ± 6	26 ± 5	29 ± 7	28.5 ± 7	28 ± 7
BMI ≥ 30 and < 40 kg/m ^2^	80 (23%)	34 (36%)	46 (31%)	25 (27%)	37 (26%)
BMI ≥ 40 kg/m ^2^	21 (6%)	4 (2%)	17 (12%)	9 (10%)	12 (9%)
Parity ≥ 3	20 (6%)	9 (5%)	11 (7%)	5 (5%)	13 (9%)
Nicotine	10 (3%)	2 (1%)	8 (5%)	2 (2%)	6 (4%)
Severe varicose veins	4 (1%)	1 (0.5%)	3 (2%)	3 (3%)	1 (0.7%)
Twin pregnancy	4 (1%)	0	4 (3%)	1 (1%)	0
ART	12 (4%)	4 (2%)	8 (5%)	3 (3%)	7 (5%)
Previous history of VTE	91 (27%)	0	91 (62%)	91 (100%)	16 (11%)
Family history of VTE	96 (28%)	48 (24%)	48 (33%)	21 (23%)	24 (17%)
History of pregnancy complications	141 (41%)	101 (52%)	40 (27%)	14 (15%)	141 (100%)
High-risk risk thrombophilia	24 (7%)	0	24 (16%)	10 (11%)	9 (6%)
Low-risk thrombophilia	97 (28%)	56 (29%)	41 (28%)	17 (19%)	30 (21%)
Antiphospholipid antibodies	29 (8%)	28 (14%)	1 (0.7%)	1 (0.1%)	28 (20%)
OAPS	11 (3%)	11 (14%)	0	0	11 (8%)
OMAPS	3 (0.8%)	3 (1.5%)	0	0	3 (2%)
NC-OAPS	14 (4%)	14 (7%)	0	0	14 (10%)
TAPS	1 (0.3%)	0	1 (0.7%)	1 (0.1%)	0
Antithrombotic treatment	238 (70%)	109 (56%)	129 (88%)	87 (95%)	115 (82%)
LMWH	155 (45%)	53 (27%)	102 (69%)	75 (82%)	53 (38%)
ASA	27 (8%)	21 (11%)	6 (4%)	1 (1%)	19 (13%)
LMWH + ASA	56 (16%)	35 (18%)	21 (14%)	11 (12%)	43 (30%)
None	104 (30%)	86 (44%)	18 (12%)	4 (4%)	26 (18%)

Notes: VTE risk stratification is based on the RCOG score 2015 11 : low risk 1–2, intermediate risk 3 and high risk > 3 antepartal points. The most important risk factor is a previous history of VTE (3 points according to the RCOG score).

Abbreviations: APS, antiphospholipid syndrome; ART, assisted reproductive technology; ASA, acetylsalicylic acid; BMI, body mass index; LMWH, low-molecular- weight heparin; NC-OAPS, noncriteria OAPS; OAPS, obstetric APS; OMAPS, obstetric morbidity APS; RCOG, Royal College of Obstetricians and Gynaecologists; SD, standard deviation; TAPS, thrombotic APS; VTE, venous thromboembolism.

A total of 141 of 342 patients (41%) were noted to have a history of pregnancy complications, whereas 91 (27%) had a personal and 96 (28%) reported a family history of VTE. Patients with pregnancy complications had a history of one or more miscarriage ( *n* = 113, including 34 patients with recurrent pregnancy loss), intrauterine fetal death (IUFD) ( *n* = 17), preeclampsia ( *n* = 12), placental insufficiency ( *n* = 11), placental abruption ( *n* = 5), or intrauterine growth restriction ( *n* = 5).

High- and low-risk thrombophilia were defined according to the criteria of the Royal College of Obstetricians and Gynaecologists (RCOG) 10 : low-risk thrombophilia diagnoses included heterozygous FVL or PGM, whereas high-risk thrombophilia included AT, PC, and PS deficiency, as well as compound heterozygosity or homozygosity for FVL and/or PGM. We detected inherited thrombophilia risk factors in 121 of 342 (35%) individuals, including 97 with low-risk thrombophilia (heterozygous FVL *n* = 78, and heterozygous PGM *n* = 19) and 24 with high-risk thrombophilia: AT deficiency *n* = 1; PC deficiency *n* = 13; homozygous FVL *n* = 3; six patients carried a heterozygous FVL mutation, of which two were combined with heterozygous PGM, one was combined with PC deficiency, and three were combined with PS deficiency. One additional patient was found to have a homozygous FVL in combination with heterozygous PGM.

The mean RCOG score was 2; 195 of 342 (57%) subjects had an RCOG score of <3 and 147 of 342 (43%) had an RCOG score ≥ 3. The prevalence of low-risk thrombophilia was 56 of 195 (29%) and 41 of 147 (28%) with an RCOG score <3 and ≥3, respectively. Antiphospholipid antibodies were present in 29 of 342 (8%) patients. Among these, patients with antiphospholipid syndrome (APS) could be further subclassified as obstetric APS (OAPS) ( *n* = 11, 3%), obstetric morbidity APS (OMAPS) ( *n* = 3, 0.8%), noncriteria OAPS ( *n* = 14, 4%), and thrombotic APS (TAPS) ( *n* = 1, 0.3%).

Antithrombotic treatment was prescribed in 238 of 342 (70%) individuals with either low-molecular-weight heparin (LMWH) ( *n* = 155, 45%), acetylsalicylic acid (ASA) ( *n* = 27, 8%), or both ( *n* = 56, 16%). LMWH was more often administered in patients with an RCOG score ≥ 3 ( *n* = 123 of 147, 84%) than in patients with a score < 3 ( *n* = 88 of 195, 45%). Nearly all patients with a personal history of VTE were treated with heparin ( *n* = 86 of 91, 95%), the five untreated patients had a history of superficial vein thrombosis (SVT) and thus did not require antepartal LMWH. Of the 141 patients with a history of pregnancy complications, 53 (38%) were treated with LMWH, 19 (13%) with ASA, and 43 (30%) with both. All 29 patients with APS were administered antithrombotic therapy, including 6 (21%) with LWMH, 6 (21%) with ASA, and 17 (58%) with both.

### Calculating Reference Ranges in Pregnancy

We calculated reference intervals for coagulation and fibrinolysis markers (shown as median, 2.5 ^th^ , 75 ^th^ , and 97.5 ^th^ percentiles) at various time points during pregnancy and the postpartum period ( Table 2 ). Due to the lack of a statistically normal distribution for all MAM parameters and given the patient population in which they were tested, the term “reference ranges” should be more appropriately referred to as *expectancy* values. First, to ensure these expectancy values were as closely in line with the theoretically established reference ranges as possible (i.e., necessitating the removal of outliers), we excluded eight samples from patients who experienced acute adverse events (SVT, *n* = 3; deep vein thrombosis [DVT], *n* = 3; IUFD, *n* = 1; miscarriage, *n* = 1) and nine samples from four patients with twin pregnancy. For the postpartum period, we excluded six samples that were collected prior to the 21 ^st^ -day postdelivery, as a higher procoagulant activity could be expected in this period close to birth. Thus, expectancy values correspond to the late postpartum ( *n* = 84, days 24–70, median days 40 postdelivery). Second, it was necessary to identify confounding factors. We therefore investigated the impact of the RCOG score, the presence of thrombophilia, administration of antithrombotic therapy, and the gestational age on MAM levels.

**Table 2:** Coagulation and fibrinolysis markers (median, 2.5 ^th^ /75 ^th^ and 97.5 ^th^ percentiles, and ranges [minimum/maximum]) at different time points during pregnancy and late postpartum

a.							
		FM (µg/mL)	DD STA (ng/mL)	DD HS (ng/mL)	DD VIDAS (ng/mL)	F1 + 2 (pmol/L)	TAT (µg/L)
1 ^st^ trimester	*n*	230	228	231	229	228	229
	Median	4.0	350	347	294	195	2.4
	Percentile	3/5.4/41	270/488/2,151	126/519 /4,057	110/464 /3,139	89/267/540	1.8/3.3/21.7
	Range	3–150	270–10,120	55–26,491	77–9,618	39–778	0.9–60
2 ^nd^ trimester	*n*	336	333	337	333	332	331
	Median	4.5	580	726	559	322	4.4
	Percentile	3.0/6.3/118.5	270/925/3,225	296/1,086/3,286	226/814/2,596	129/421 /712	2.0/5.7/10.0
	Range	3.0–150.0	270–6,180	111–4,555	155–3,519	86–903	2.0–32.1
3 ^rd^ trimester	*n*	240	240	241	238	240	239
	Median	4.7	1,040	1,203	1,037	565	6.5
	Percentile	3.0/8.2/94.2	390/1,580/3,494	478/1,706/4,719	405/1,646/3,653	314/718/989	2.0/8.7 /14.2
	Range	3.0–150.0	290–8,490	282–6,639	263–6,889	157–1,181	2.0–21.0
Postpartum	*n*	84	84	84	82	84	84
	Median	3.7	350	345	274	243	2.1
	Percentile	3.0/4.7/44.0	270/480/1,814	91/525/1,373	120/426 /487	113/347/577	1.5/2.8/20.5
	Range	3.0–54.6	270–2,780	54–1,583	111–1,888	101–604	1.5–27.8
b.							
Exclusion of patients with		FM (µg/mL)	DD STA (ng/mL)	DD HS (ng/mL)	DD VIDAS (ng/mL)	F1 + 2 (pmol/L)	TAT (µg/L)
ASA		No	Yes	Yes	Yes	No	No
LMWH		No	No	No	Yes	No	No
Low-risk thrombophilia		Yes	Yes	Yes	Yes	Yes	Yes
1 ^st^ trimester	*n*	159	103	103	48	158	159
	Median	4.1	340	342	290	186	2.3
	Percentile	3/5.5/16.2	270/460/2,434	94/492/3,715	99/413/1,427	73/252/542	1.8/3.1/21
	Range	3–150	270–5,180	55–4,607	86–1,524	39–778	1–60
2 ^nd^ trimester	*n*	238	161	163	29	235	234
	Median	4.3	510	629	615	306	3.9
	Percentile	3/5.7/37.4	270/755/2,387	293/1,001/2,381	295/890/NC	123/391/647	2/5.1/10
	Range	3–132	270–2,800	189–4,025	295–2,629	86–903	2–32
3 ^rd^ trimester	*n*	155	113	113	18	154	153
	Median	4.5	840	1,068	1,008	541	6.1
	Percentile	3/7/59	377/1,310/2,597	476/1,484/4,069	510/1,656/NC	282/646/992	2/7.4/14.4
	Range	3–133	290–8,490	417–6,257	510–4,451	198–1,115	2–16
Postpartum	*n*	56	52	52	3	56	56
	Median	3.7	330	310	303	214	2
	Percentile	3/4.7/52	270/448/1,804	96/494/1,340	152/NC/NC	117/350/560	1.5/2.7/24.8
	Range	3–54.6	270–1820	87–1393	152–546	113–584	1.5–27.8
c.							
Exclusion of patients with		FM (µg/mL)	DD STA (ng/mL)	DD HS (ng/mL)	DD VIDAS (ng/mL)	F1 + 2 (pmol/L)	TAT (µg/L)
ASA		No	Yes	Yes	Yes	No	No
LMWH		No	No	No	Yes	No	No
1 ^st^ trimester	*n*	64	54	55	24	62	62
	Median	4.0	360	364	377	222	2.7
	Percentile	3/5.3/58.7	270/485/1,811	148/541/1,968	177/827/NC	97/273/536	1.9/4.1/26
	Range	3–117	270–2,040	148–2,274	177–2,617	96–636	1.8–30
2 ^nd^ trimester	*n*	85	68	68	29	84	84
	Median	4.8	705	881	654	359	5.3
	Percentile	3/10.5/147.4	270/1,295/5,201	248/1,471/4,103	252/860/NC	167–733	2/6.5/15.6
	Range	3–150	270–6,180	171–4,555	252–2,822	165–738	2–20
3 ^rd^ trimester	*n*	75	61	62	26	76	76
	Median	5.4	1,420	1,510	1,265	662	7.4
	Percentile	3/15/150	492/2,055/4,813	587/2,368/5,715	509/1,618/NC	386/807/1039	2.3 /10.2/15.0
	Range	3–150	470–5,990	564–6,639	509–3,510	358–1,181	2–21
Postpartum	*n*	24	23	23	7	24	24
	Median	3.4	370	411	346	256	2.3
	Percentile	3/4.5/NC	270/540/NC	127/554/NC	142/467/NC	101/332/NC	2/3.0/NC
	Range	3–15.5	270–2,780	127–1,583	142–862	101–604	2–3.5
d.							
Exclusion of patients with		FM (µg/mL) all patients			Only patients with low-risk thrombophilia		
Adverse events, twin pregnancy		Yes	Yes		Yes		
ASA		No	No		No		
LMWH		No	No		No		
Low-risk thrombophilia		Yes	No		No		
All trimesters and late puerperium	*n*	608	856		248		
	Median	4.2	4.3		4.5		
	Percentile	3.0/5.8/40.0	3.0/6.3/84.2		3.0/7.5./132.5		
	Range	3.0–150	3.0–150		3.0–150		
1 ^st^ –3 ^rd^ trimester	*n*	552	776		224		
	Median	4.3	4.4		4.7		
	Percentile	3.0/5.9/37.9	3/6.5/92.8		3/8/132.9		
	Range	3.0–150	3.0–150		3.0–150		

Abbreviations: ASA, acetylsalicylic acid; DD, D-dimer; F1 + 2, prothrombin fragments 1 and 2; FM, fibrin monomer; LMWH, low-molecular-weight heparin; NC, not calculable; PT, prothrombin time; STA, Stago; TAT, thrombin–antithrombin complex.

Notes: (a) All patients. (b) Patients without adverse events, twin pregnancy, low-risk thrombophilia, and with (FM, F1 + F2, TAT) and without (DD) antithrombotic treatment. (c) Patients with low thrombophilia, but without adverse events, twin pregnancy, high-risk thrombophilia, and with (FM, F1 + F2, TAT) and without (DD) antithrombotic treatment. (d) FM calculated irrespective of trimester/postpartum period (patients without adverse events, twin pregnancy) with and without low-risk thrombophilia.

**Fig. 1:**
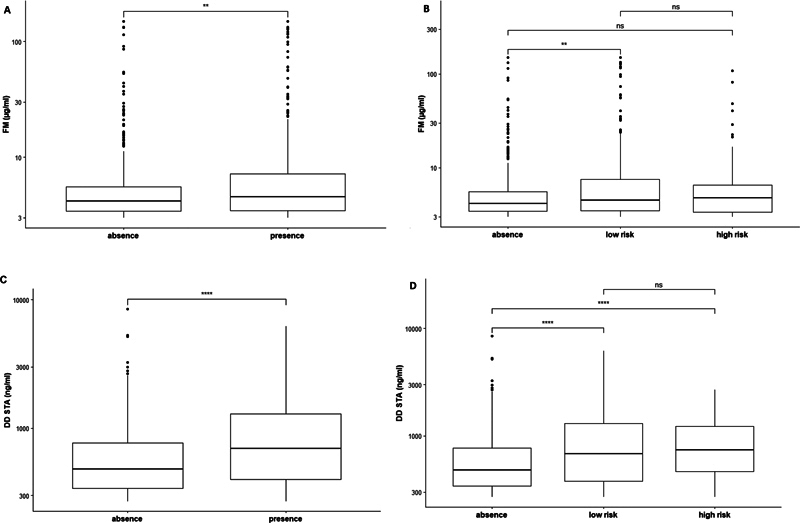
Association between thrombophilia (low- and high-risk) and fibrin monomer (FM) and D-dimer (DD) levels: ( **A** ) FM levels: absence vs. presence of thrombophilia ( **B** ) FM levels: absence vs. low- and high-risk thrombophilia ( **C** ) DD levels: absence vs. presence of thrombophilia ( **D** ) DD levels: absence vs. low- and high-risk thrombophilia. Each box represents the interquartile range (IQR), which is between the 25 ^th^ and 75 ^th^ percentile. The central line represents the median. Upper/lower whiskers indicate “Q3 + 1,5*IQR” and “Q1 – 1.5*IQR”, respectively. Abbreviation: ns: non-significant. * *p* < 0.05; ** *p* < 0.01, *** *p* < 0.001; **** *p* < 0.0001 according to the Wilcoxon test.

No correlation was found between the RCOG score and any of the MAM levels. In patients with an RCOG score ≥ 3, treatment with ASA showed a slight ( *p* = 0.013 for DD STA and DD HS) and strong ( *p* = 0.004 for DD VIDAS) impact on DD levels, respectively. LMWH treatment only slightly affected DD levels, as determined by DD VIDAS ( *p* = 0.025). Levels of F1 + 2, TAT, and FM were unaffected by any antithrombotic treatment. Significantly higher levels of FM ( *p* < 0.01) and DD STA ( *p* < 0.0001) were detected in the presence versus absence of inherited thrombophilia ( Fig. 1 ). Thus, for the purpose of calculating expectancy values we further excluded all samples with low-risk thrombophilia ( *n* = 253) for all MAM as well as samples with LMWH treatment for the DD VIDAS assay only ( Table 2B ). This resulted in a smaller sample size; thus, the 97.5 ^th^ percentiles for DD VIDAS from the second trimester and postpartum period were not calculable (NC). The presence of antiphospholipid antibodies in OAPS, OMAPS, and NC-APC had no influence on any MAM. TAPS appeared to be associated with higher levels of MAM; however, this group consisted of only four samples from a singular patient, so this observation could not be statistically substantiated.

**Fig. 2:**
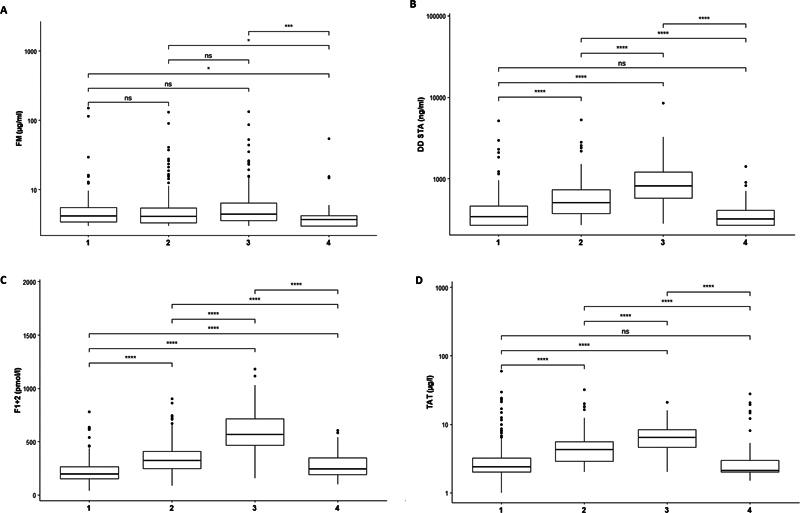
Course of fibrin monomer (FM), D-dimer (DD), prothrombin fragments (F1+2) and thrombin-antithrombin complex (TAT) at different timepoints during pregnancy first: (1), second (2) and third (3) trimester and the postpartum period (4). The trimester does not impact FM levels except for the post-partum period ( **A** ), but impacts DD levels ( **B** ), F1+2 ( **C** ) and TAT levels ( **D** ). Each box represents the interquartile range (IQR), which is between the 25 ^th^ and 75 ^th^ percentile. The central line represents the median. Upper/lower whiskers indicate “Q3 + 1,5*IQR” and “Q1 – 1.5*IQR”, respectively. Abbreviation: ns: non-significant. * *p* < 0.05; ** *p* < 0.01, *** *p* < 0.001; **** *p* < 0.0001 according to the Wilcoxon test.

We found steady and significant increases of the markers DD, F1 + 2, and TAT from the beginning to the end of pregnancy, with each subsequent trimester showing higher levels that than the last. On the other hand, there was no association between FM level and gestational age or pregnancy trimester (except for the postpartum period) ( Fig. 2 ). Thus, expectancy values for FM could be calculated independently from the trimester ( Table 2D ). The last column of Table 2D presents FM expectancy ranges exclusively for pregnant patients with low-risk thrombophilia. For DD, F1 + 2, and TAT, we calculated expectancy values trimester-wise ( Table 2B ).

**Fig. 3:**
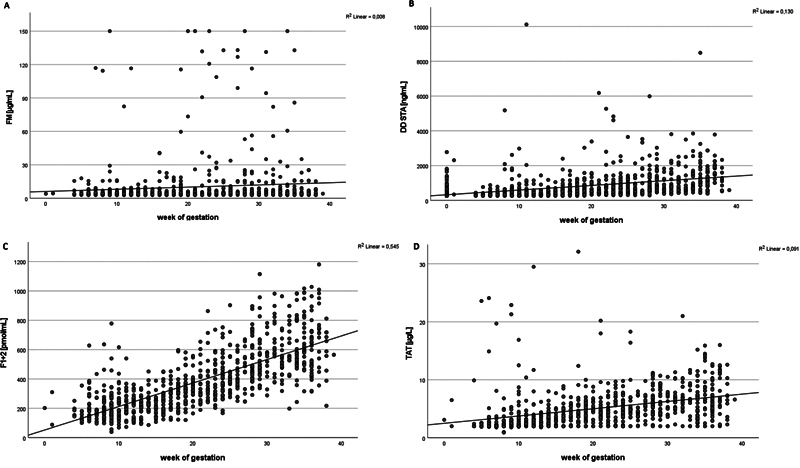
Correlations between gestational age (x-axis) and fibrinolysis marker (Y-axis) for ( **A** ) FM, ( **B** ) DD (STA), ( **C** ) F1+2 and ( **D** ) TAT (each Y-axis) for all patients.

**Fig. 4:**
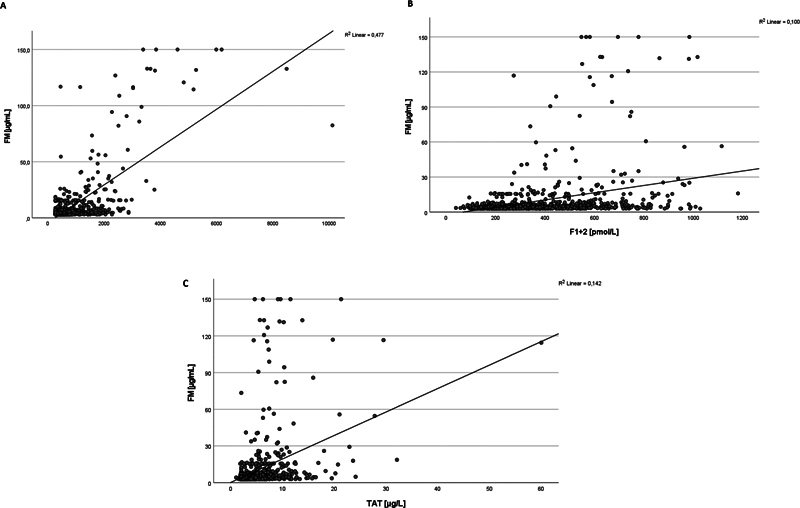
Interassay correlations of fibrinolysis markers for FM (Y-axis) and ( **A** ) DD (STA), ( **B** ) F1+2 and ( **C** ) TAT (each x-axis).

Table 2C shows expectancy values for all MAM in pregnancy and postpartum exclusively for patients with low-risk thrombophilia. Gestational age was strongly correlated with F1 + 2 (0.738) and weakly for DD STA and TAT (0.356 and 0.301, respectively). For FM, there was no correlation (0.087) ( Fig. 3 ). The interassay correlations for FM versus DD were all significantly higher for FM versus DD STA (0.691), FM versus DD HS (0.533), and FM versus DD VIDAS (0.505), and lower for FM versus F1 + 2: FM (0.317) and FM versus TAT (0.377) ( Fig. 4 ). Among the various DD assays, we found the highest interassay correlations using two-tailed Pearson's correlations ( *p* < 0.001) for DD STA versus DD HS (0.898), DD STA versus DD VIDAS (0.871), and DD HS versus DD VIDAS (0.901).

### Cases with Outlier of Fibrin Monomer Levels

Clinical details of cases with outlier of FM levels (cases 1–8) are shown in the Supplementary Data . Acute DVT occurred in two patients (cases 1 and 2), SVT in three patients (cases 3, 4, and 5), and miscarriage in three further patients (cases 6, 7, and 8). This corresponds to 0.6, 0.9, and another 0.9% of all pregnancies, respectively. FM levels > 37.9 µg/mL were found in 37 samples (4%) from 20 patients. The incidence of DVT in these samples was 10%. In samples with single pregnancy and without adverse events, we detected FM levels > 37.9 µg/mL in 34 samples (4%) from 18 women, among them 19 (55%) and 4 (12%) with low- and high-risk thrombophilia, respectively.

## Discussion

The aim of our study was to establish reference ranges for FM levels in pregnancy and the postpartum period. However, as opposed to establishing reference intervals in “normal donors,” our data were derived from patients seen at our ambulatory hemostasis clinic, where they were evaluated for an elevated risk of VTE and/or vascular pregnancy complications. These patients presented with various thrombophilic risk profiles and with or without antithrombotic treatment. We therefore suggest replacing the term “reference values” with “expectancy values in uncomplicated pregnancy.” For the purpose of this study, we excluded patients who experienced acute adverse events as well as twin pregnancies, as these may impact coagulation activation markers. In addition, it was necessary to identify other factors that could have a relevant impact on expectancy values and to exclude these samples as well.

### Exclusion of Patients with Adverse Events

As expected, in two patients who presented with acute DVT in pregnancy (cases 1 and 2) all MAM were found to be elevated. However, in one patient with SVT, FM was decreased in contrast to DD, which was found to be in the upper expectancy range to slightly increased, depending on the assay used (case 4). In another patient who experienced a miscarriage in the 9 ^th^ week of gestation (case 6), the FM level was elevated in contrast to DD, F1 + 2, and TAT, which all fell within the expected range. Due to the low incidence of DVT (0.6%), SVT (0.9%), and miscarriage (0.9%) in our study, no definite conclusions can be drawn regarding the clinical utility of FM analysis in pregnancy in terms of determining VTE risk. FM may be a promising candidate due to the stable course of FM levels throughout pregnancy, in contrast to other markers such as DD, F1 + 2, and TAT ( Fig. 3 ). Onishi et al previously suggested FM as a possible prognostic thrombotic marker for VTE and defined a cutoff value at 24.4 mg/L for VTE diagnosis, using the Auto LIA FM kit. 17 In our study using the STA-LIATESTFM we observed only two cases with acute DVT exhibiting FM levels of 82 and ≥150 ng/mL, respectively. Therefore, our limited data were not sufficient for calculating a cutoff for diagnosing VTE in pregnancy. However, FM remains a promising biomarker with the potential to provide additional information on actual thrombus formation, and therefore, FM could be more predictive for VTE diagnosis and exclusion in pregnancy. Further prospective studies are needed to investigate this as well as whether this could also apply to pregnancy complications (such as miscarriage or preeclampsia).

The low incidence of DVT in our cohort, which is within the general rate in pregnancy, 6 24 25 is probably related to the high proportion of patients receiving antithrombotic therapy (70%) as part of their clinical management. Of note, 39% of patients with an RCOG score below three points received LMWH, indicating that prescribing heparin in pregnancy is not exclusively dependent on the individual VTE risk. A total of 71% of the patients receiving LMWH injections despite a low RCOG score reported a history of pregnancy complications, which may have been related to the high proportion of patients in this group (36%) who were found to have positive antiphospholipid antibodies ( Table 1 ). However, the rate of (noncriteria) OAPS in this cohort does not fully explain this observation, and thus, other factors such as the goal of maintaining pregnancy or reducing the risk of pregnancy complications (in patients with a prior history) may be considered when weighing the risks and benefits of prescribing LMWH in pregnancy. In other words, the benefits of LMWH prophylaxis are not only a matter of the individual's VTE risk, but also have to do with the maintenance of a high-risk pregnancy and the possible requirement for extra precautionary measures.

### Influence of Venous Thromboembolism Risk and Low Thrombophilia

We detected no correlation between MAM levels and VTE risk assessed by the RCOG score, which is in accordance with the results of previous studies stratifying pregnant women into low- and high-risk VTE groups. 26 27 However, one recent study found higher DD and FM concentrations in patients with RCOG scores ≥ 3 compared with scores < 3: DD 4,500 versus 2,600 ng/mL and FM 14.6 versus 3.4 µg/mL, respectively. 19 Although low-risk thrombophilia (heterozygosity for FVL or PGM) is a part of the RCOG score, it seems to influence the levels of all MAM. In the aforementioned study, data regarding the impact of inherited thrombophilia in both groups are lacking. 19 Importantly, in our study the prevalence of low-risk thrombophilia was similar in both groups (RCOG score ≥ 3 vs. <3) with 28 and 29%, respectively.

Therefore, for calculating expectancy values we excluded patients with low-risk thrombophilia, as this might influence all MAM levels ( Table 2B and D ). This finding indicates that low-risk thrombophilia has a more pronounced effect on hemostasis than acquired, exogenous, or other dispositional thrombophilic risk factors. A study by Elmas et al demonstrated that injection of endotoxin led to a greater increase in soluble fibrin in patients with FVL than in controls. 28 Simioni et al could already demonstrate in 1996 increased F1 + 2 and TAT levels in carriers of FVL, 29 but this observation could not be confirmed in a Greek study 1 year later. 30 In addition, Rühl et al observed after in vivo coagulation activation by recombinant factor VIIa a higher increase of F1 + 2 and TAT levels in carriers of FVL and PTM as compared with healthy controls. 31 Furthermore, it has been suggested that the fibrinolytic response differs in thrombophilia patients depending on the underlying thrombophilia risk factors, which in turn modulates the risk of thrombosis. 32 Patients with the FVL mutation also displayed higher levels of DD and FDP in plasma after 24 hours, as factor Va activity is 10-fold in carriers of heterozygous FVL in contrast to noncarriers. Paidas et al reported higher levels of soluble fibrin polymer in the first trimester of pregnancy in patients with thrombophilia as compared with controls. 33 It remains important to note that in our study low-risk but not high-risk thrombophilia had an impact on all activation markers in pregnancy, which seems to be counterintuitive but may be explained by the lower sample size in the high-risk thrombophilia group ( Fig. 1 ).

### Influence of Antithrombotic Treatment

In our study levels of F1 + 2, TAT, and FM were unaffected by antithrombotic treatment during all stages of pregnancy and the postpartum period. However, as our findings suggest an influence of ASA on all DD tests, we excluded samples of patients treated with ASA for calculating the expectancy ranges of DD. Treatment with LMWH only showed an impact on DD levels, if they were assessed by VIDAS. This could be related to differences between the epitopes recognized by the individual monoclonal antibodies in the large DD antigen. 34 This also raises the question of whether the DD assay selectively recognizes (and measures) the “small” DD structure with a molecular weight of 180 kDa, or whether it can also detect the larger DD structure that is already formed within soluble fibrin crosslinked in the D domains of neighboring D domains, covalently linked by factor XIIIa. Another explanation could be that the antibodies in the assay are picking up larger soluble FDP. The difference in the effects on DD testing between ASA and LMWH could be due to the inhibition of fibrin formation by LMWH, whereas ASA neither inhibits the fibrinogen–fibrin conversion nor the subsequent crosslink of FMs by factor XIIIa. Neither ASA nor LMWH therapy had an impact on FM levels, which again supports the hypothesis that FM is more robust as a biomarker and subject to little influence. The significant interassay correlation ( Fig. 4 ) indicates that FM is dependent on hemostasis activation in pregnancy as reflected by the large number of unexplained “outliers” ( Fig. 3 ). However, stable FM levels may be indicative that the hypercoagulable physiological shift in pregnancy is well controlled. Therefore, instead of the widespread approach of serial DD testing for evaluating VTE risk in pregnancy, an adapted regimen involving FM testing appears to be more promising, as this parameter provides an additional information about intravascular in vivo clot formation. Moreover, the negative predictive value of FM may be more useful for the exclusion of suspected VTE in pregnancy and the postpartum period.

### Challenges and Limitations of Establishing Reference Values in Pregnancy

Establishing reference values in pregnancy and the puerperium is challenging for several reasons, a fact that is reflected by the lack of clear consensus guidelines for hemostatic biomarker testing and interpretation. Tang et al concluded in their meta-analysis that there are still no universal hemostatic reference ranges during pregnancy and postpartum. 35 In previous studies concerning FM levels, patients with pregnancy complications were excluded 17 18 20 21 22 or considered “healthy” if they had no personal or family history of VTE. 20 23 Interestingly, Grossmann et al excluded patients on anticoagulation 22 and Joly et al excluded those with any form of antithrombotic treatment. 20 None of these studies explicitly excluded patients with thrombophilia, and in a study by Kristoffersen et al, these patients were noted to be present. 23 Information about antithrombotic treatments and VTE risks was absent in a study by Kawamura et al 21 ; however, for FM (STA-R), they considered an FM level of ≥35 µg/mL to be as abnormal, which is in accordance with our result of an FM level of 37.9 µg/mL falling in the 97.5 ^th^ percentile. In their study, patients were excluded if they had not undergone all scheduled examinations. In our present study, we did not exclude these patients, since the time of blood collection was not predetermined by scheduled study visits, but rather performed during each routine visit to our hemostasis clinic, the timing of which varied from patient to patient. This was reflected in the low examination rate of 2.6 per pregnancy. In addition, Kawamura et al found that 8.4% had values over the cutoff limit of ≥35 µg/mL as compared with our study, in which 5.8% had levels > 37.9 µg/mL. Kawamura et al performed compression ultrasonography of the lower extremities in the 50 women with abnormal FM levels and detected thrombi in three of these patients (6%). The proportion of patients with FM values > 37.9 µg/mL and DVT/SVT in our study was determined to be 1%, although we did not systematically perform compression ultrasonography, instead offering clinical monitoring of signs and symptoms of VTE. The lower proportion of abnormal FM levels compared with other MAM affirms the hypothesis that FM reflects more accurate intravascular in vivo clot formation; thus, FM may also have a better negative predictive value for diagnosing DVT in pregnancy.

In another study, Joly et al investigated FM concentration with the most restrictive selection criteria, having excluded patients with hypertension, gestational diabetes, and abnormal F1 + 2 and DD levels in addition to the aforementioned features. 20 They calculated mean and SD FM levels for each trimester and observed significant differences between the first and second, and second and third trimesters. In contrast, we found no association between the gestational trimester and FM levels, except for during the postpartum period ( Fig. 2 ). The absence of any increase of F1 + 2, TAT, and DD levels in the postpartum period is mainly explained by the fact that postpartum blood collection during this period was performed late (median day 40 postdelivery), by which time the MAM levels were most likely returning to baseline. More precisely, the postpartum intervals recorded in our study rather reflect the late puerperium (>21 days after delivery).

## Conclusions

We observed various changes in MAM levels throughout pregnancy and the late postpartum period: while DD increases significantly during pregnancy, this was not quite as pronounced as for F1 + 2 and TAT. In contrast, the marginal increase in FM levels during pregnancy reflects low or absent intravascular fibrin formation. The presence of a low-risk thrombophilia was found to influence all MAM levels in pregnancy. Except for DD (assessed by VIDAS), LMWH treatment during pregnancy had no effect on MAM. Given the low incidence of DVT in our study cohort, the predictive value of elevated markers of fibrinolysis was not assessed. Therefore, we do not recommend routine, serial MAM testing in pregnant patients with low and intermediate VTE risk. However, measuring FM (together with DD, F1 + 2, and TAT) could be an additional tool for screening pregnant patients with high VTE risk and those with suspicion of VTE. The expected upper reference range for FM concentration in pregnant patients without low-risk thrombophilia was found to be 37.9 µg/mL, which may differ from the unknown threshold for diagnosing VTE in pregnancy. Notably, the much higher 97.5 ^th^ percentile FM level of 132.9 µg/mL in pregnant patients with low-risk thrombophilia could limit its use when solely diagnosing and ruling out VTE. Due to the sample size, our data did not allow for the calculation of a clinically useful cutoff value, nor any negative and positive predictive values for FM. This should be further investigated in future prospective studies involving pregnant and nonpregnant patients with suspected DVT following ultrasound examination.

## Notes

**What is Known about This Topic?**

- Markers of coagulation activity and fibrinolysis such as DD physiologically increase during pregnancy and are dependent on several dispositional (e.g., thrombophilia, age, BMI) and expositional risk factors (e.g., ovarian hyperstimulation syndrome, hyperemesis, immobilization).
- Establishing reference values for coagulation markers in pregnancy is still challenging due to the heterogeneity of the pregnant population in terms of VTE risk factors. The predictive value of DD is limited regarding determining VTE risk or diagnosing/excluding VTE in pregnancy.
- FM levels remain stable during pregnancy and thus could have potential as a clinically useful biomarker for coagulation and fibrinolysis activation.

**What Does This Paper Add?**

- The presence of hereditary low-risk thrombophilia (such as heterozygosity for FVL or prothrombin G20210A mutation) seems to have a greater effect than acquired thrombophilia or other dispositional risk factors on procoagulant activity in pregnancy.
- FM concentration is influenced by low-risk thrombophilia but not antithrombotic treatment. The upper reference interval in pregnancy could be calculated, regardless of the trimester and was found to be 37.9 µg/mL.
- Further studies are needed to establish a cutoff FM value for diagnosing VTE in pregnancy, as FM could have clinical utility for evaluating VTE and VTE risk in lieu of DD.
