# Supplementary material for: Establishing Expectancy Values for Fibrin Monomer in Uncomplicated Pregnancy
Source: TH Open. 2024 Jul 11;8(3):e283–96. doi: 10.1055/s-0044-1788281 (PMC11239220; doi:10.1055/s-0044-1788281)
Supplement: Supplementary file 1 — Supplementary Material [file 10-1055-s-0044-1788281-s24060020.pdf]

Supplementary Data

Case 1

A 31-year-old nulliparous female with bronchial asthma was referred in the 9th and 17th week of gestation due to a known heterozygous factor V Leiden mutation and a family history of venous thromboembolism. In the 23rd week, she reported increased blood pressure values and dyspnea that persisted for 2 to 3 weeks. In the timeframe between the 17th and 23rd gestational week, D-dimer (DD) HS rose from 1,319 to 4,268 ng/mL and the fibrin monomer (FM) level increased from 19 to $\geq$ 150 µg/mL. Duplex ultrasound revealed an acute distal fibular vein thrombosis. A pulmonary embolism was considered unlikely, which was supported by a normal echocardiography and a negative troponin test. Anticoagulation was initiated with 8.000 U enoxaparin subcutaneously twice daily and continued until 6-week postpartum. She delivered spontaneously at the 40 weeks + 3 days of gestation (estimated blood loss 400 mL).

Case 2

A 26-old-year old nulliparous patient was referred in the 8th week of gestation due to a known hereditary antithrombin (AT) deficiency type 1 (AT activity 42% [reference range: 83–128%]), low AT antigen level of 48% [reference range: 80–120%]) and a family history of pregnancy-related deep vein thrombosis (DVT) in her mother at the age of 23. After starting thromboprophylaxis with 4.000 U enoxaparin subcutaneously once daily since the 7th week, she developed a DVT on the left side after a muscle strain. DD HS increased from 412 to 10,859 ng/mL (8th and 11th gestational week, respectively) and FM levels were elevated at 82 µg/mL at week 11 of gestation. Therapeutic anticoagulation was initiated with 6.000 U enoxaparin subcutaneously twice daily, which continued postpartum (spontaneous delivery at 40 weeks). She continued with 6.000 U enoxaparin subcutaneously administered once daily for 3 more months while breastfeeding.

Case 4

A 32-year-old multiparous patient with varicose veins and history of posttraumatic superficial vein thrombosis (SVT) in the 21st week of gestation in a prior pregnancy presented herself with painful swelling of her right thigh. She presented in the 9th week of her fifth pregnancy and reported no additional thrombophilic risk factors. Ultrasound confirmed the diagnosis of SVT. Low-molecular-weight heparin (LMWH) prophylaxis with 4.000 IU enoxaparin subcutaneously once daily was initiated. FM level was low (8.21 µg/mL), but DD levels were in the upper range on DD STA and DD HS (1,740 and 2,127 ng/mL, respectively) and slightly increased DD VIDAS (2,250 ng/mL).

Case 6

A 28-year-old obese patient (body mass index: 35 kg/m^2) with a history of intrauterine fetal death (IUFD) in the 27th week of gestation in her first pregnancy was started on acetylsalicylic acid 100 mg once daily in her second pregnancy and presented to our clinic in the 7th gestational week. In the presence of repeated weak IgG antibodies against annexin V, LMWH prophylaxis was initiated with dalteparin 5.000 IU subcutaneously once per day, but the patient suffered a miscarriage in the 9th week of gestation. The examination during the 7th week revealed normal DD (DD STA 450 ng/mL, DD HS 176 ng/mL, and DD VIDAS 220 ng/mL, respectively) and F1 + 2 (273 pmol/L), but an elevated FM level (117 µg/mL), whereas the level of TAT was with 19.7 µg/L in the upper expectation range.

In cases 3, 5, 7, and 8 FM levels were lacking at the time the adverse event occurred: SVT (case 3), postpartum SVT (case 5), miscarriage > 12th week (case 7), and IUFD due to placental insufficiency and early abortion (case 8).

 © 2024. The Author(s).
